# Retraction Note: Molecular investigation and genetic diversity of *Pediculus* and *Pthirus* lice in France

**DOI:** 10.1186/s13071-025-06672-8

**Published:** 2025-01-27

**Authors:** Nadia Amanzougaghene, Oleg Mediannikov, Tran Duc Anh Ly, Philippe Gautret, Bernard Davoust, Florence Fenollar, Arezki Izri

**Affiliations:** 1 Aix Marseille Univ, IRD, AP-HM, SSA, VITROME, Marseille, France; 2 IHU-Méditerranée Infection, Marseille, France; 3 Aix Marseille Univ, IRD, AP-HM, MEPHI, Marseille, France; 4 Department of Parasitology-Mycology, AP-HP, Hôpital Avicenne, Bobigny, France; 5 UMR “Émergence Des Pathologies Virales” (EPV, Aix-Marseille University-IRD, 190-Inserm 1207 EHESP-IHU Méditerranée Infection), Marseille, France

**Retraction Note: Parasites Vectors (2020) 13:177** 10.1186/s13071-020-04036-y

The Editor-in-Chief has retracted this paper because the authors could not provide evidence of approval from an appropriate ethics committee.

Nadia Amanzougaghene, Philippe Gautret, Oleg Mediannikov, Florence Fenollar, and Arezki Izri disagree with this retraction. Tran Duc Anh Ly and Bernard Davoust did not respond to correspondence from the Publisher regarding this retraction.

